# Nematicidal Activity of Benzyloxyalkanols against Pine Wood Nematode

**DOI:** 10.3390/biom11030384

**Published:** 2021-03-05

**Authors:** Junheon Kim, Su Jin Lee, Joon Oh Park, Kyungjae Andrew Yoon

**Affiliations:** 1 Forest Insect Pests and Disease Division, National Institute of Forest Science, Seoul 02455, Korea; sujin0316@inu.ac.kr (S.J.L.); joonoh6@gmail.com (J.O.P.); 2 Research Institute of Agriculture and Life Sciences, Seoul National University, Seoul 08826, Korea; kongbob89@snu.ac.kr

**Keywords:** pine wood nematode, pine wilt disease, benzyloxyalkanol, mode of action

## Abstract

Pine wilt disease (PWD) is caused by the pine wood nematode (PWN; *Bursaphelenchus xylophilus*) and causes severe environmental damage to global pine forest ecosystems. The current strategies used to control PWN are mainly chemical treatments. However, the continuous use of these reagents could result in the development of pesticide-resistant nematodes. Therefore, the present study was undertaken to find potential alternatives to the currently used PWN control agents abamectin and emamectin. Benzyloxyalkanols (BzOROH; R = C_2_–C_9_) were synthesized and the nematicidal activity of the synthetic compounds was investigated. Enzymatic inhibitory assays (acetylcholinesterase (AChE) and glutathione *S*-transferase (GST)) were performed with BzOC8OH and BzOC9OH to understand their mode of action. The benzyloxyalkanols showed higher nematicidal activity than did benzyl alcohol. Among the tested BzOROHs, BzC8OH and BzC9OH showed the strongest nematicidal activity. The LD_50_ values of BzC8OH and BzC9OH were 246.1 and 158.0 ppm, respectively. No enzyme inhibitory activity was observed for BzC8OH and BzC9OH. The results suggested that benzyloxyalcohols could be an alternative nematicidal agent.

## 1. Introduction

Pine wilt disease (PWD), caused by the pine wood nematode (PWN; *Bursaphelenchus xylophilus*), causes severe environmental damage to global pine forest ecosystems [1]. PWD has become and is becoming a serious concern over multiple continents including Asia and Europe [2,3,4,5,6,7]. Since its first reported sighting in 1988 in Korea [8], it has spread nationwide and ca. 490,000 trees are affected by PWD [9]. The majority of pine species affected by PWD are red pine *(Pinus densiflora*), black pine (*P. thunbergii*) and *P. koraiensis* in Asia, while *P. pinaster*, *P. nigra* and *P. radiata* are affected in Europe [1,3,10]. As *Pinus* species are the predominant tree species in Korean forests and highly affected by PWD, the ecological and economic damage is substantial.

The current strategies used to control PWN are mainly chemical treatments, which include nematicide injection in trunks for PWD prevention and fumigation of wood material. The most frequently used nematicides in Korea are abamectin and emamectin benzoate, which belong to the avermectin family [11,12]. These agents are known to be highly effective against PWN and are also considered environmentally safe [13,14]. However, it could be inferred that the continuous use of these nematicides will induce selection pressure and almost certainly produce resistant PWNs, as has been reported previously for nematodes that developed resistance to avermectins [15,16,17].

As nematicides against PWN are applied in closed systems, such as trunk injection, the possibility of developing resistance against avermectins is low and opportunities for selected nematode strains to spread in natural populations is limited. However, because the nematicidal effect of avermectins is not always successful, the possibility of developing resistance and spreading by insect vectors in natural populations remains. Although PWN has not yet been reported to show resistance to abamectin, the alternative use of diverse agents is recommended to avoid the development of resistance and to achieve efficient control of PWN. In recent decades, as PWN control agents, the use of microorganism extracts, plant extracts, essential oils and volatiles has been suggested [18,19,20,21,22,23].

It has been reported that metabolic resistance in insects involves some detoxifying enzymes which are responsible for breaking down toxins entering their bodies [24]. Among those enzymes, acetylcholinesterase (AChE) and glutathione *S*-transferase (GST) have been assessed to obtain an understanding of the mode of action and the extent of its detoxification in the target species [24,25,26]. Inhibition of AChE causes overstimulation of the neurons, twitching of muscles and insect death [27] and GST is involved in resistance development in insects through detoxifying of toxins [28].

Recently, the nematicidal effect of alkyloxyethanols (ROR’OH, R’ = ethyl), which modified hydroxy groups (-OH) to ɷ-hydroxyalkyl groups (-OROH) in aliphatic compounds, against PWN was investigated [29]. The effect of modification of the modified hydroxy group to the ɷ-hydroxyalkyl group in aromatic compounds has not been investigated in PWN. In the present study, we synthesized benzyloxyalcohol (BzOROH; R = C_2_–C_9_), evaluated the nematicidal effects of 8 benzyloxyalcohols and investigated their mode of action using AChE and GST inhibition assays.

## 2. Materials and Methods

### 2.1. Nematodes

*Bursaphelenchus xylophilus* was provided by the National Institute of Forest Science, South Korea. The identification of *B. xylophilus* was confirmed using morphological characteristics and genetic differences, which were confirmed by the RFLP method [30]. The *B. xylophilus* were reared on a fungal mat of *Botrytis cinerea* on potato dextrose agar (PDA) medium at 25 ± 1 °C and 40% humidity for several generations.

### 2.2. Preparation of Benzyloxyalcohols

**Scheme 1:**
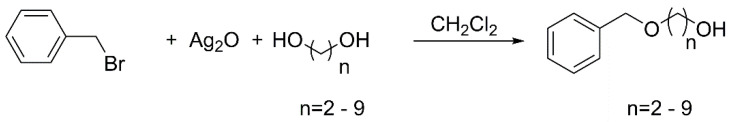
Synthetic scheme of benzyloxyalcohols.

Benzyloxyalkanols (**1**–**8**) were synthesized by modifying the method of Tanabe and Peters [31] (Scheme 1). The general procedure was as follows: a 3-necked round-bottom flask was equipped with a magnetic stirring bar, a pressure-equalizing dropping funnel, a reflux condenser, and an inlet for nitrogen. The flask was charged with silver (I) oxide (15 mmol, Alfa Aesar, Heysam, UK) and alkanediol (15 mmol; ethanediol, propanediol, butanediol, pentanediol, hexanediol, heptanediol, octanediol and nonanediol) in 30 mL of CH_2_Cl_2_ (Daejung, Hwaseong, Korea), while benzyl bromide (11 mmol, Alfa Aesar) in 10 mL of CH_2_Cl_2_ was added over ca. 10 min. The mixture was stirred for 15 h at room temperature. The suspension was filtered through Celite (Alfa Aesar), and the filter cake was washed with two portions of 50 mL of diethyl ether. The ethereal phase was washed with water and brine and dried over MgSO_4_. After filtration and evaporation, the residue was subjected to silica gel column chromatography to obtain the desired compounds (35% diethyl ether in hexane fraction). Their structure was confirmed by NMR. Their purity was determined by gas chromatography-mass spectrometry (GC-MS).

2-(Benzyloxy)-1-ethanol (BzOC2OH, **1**)

Yield 51.2%. Purity 97.1%. ^1^H NMR (500 MHz, CDCl_3_) δ (ppm) 7.386–7.320 (m, 4H), 7.314–7.233 (m, 1H), 4.558 (s, 2H), 3.749 (q, *J* = 4.9 Hz, 2H), 3.592 (t, *J* = 4.9 Hz, 2H). ^13^C NMR (126 MHz, CDCl_3_) δ (ppm) 137.97 (C), 128.47 CH × 2), 127.81 (CH × 3), 73.30 (CH_2_), 71.39 (CH_2_), 61.89 (CH_2_).

3-(Benzyloxy)-1-propanol (BzOC3OH, **2**)

Yield 93.8. Purity 95.6%. ^1^H NMR (500 MHz, CDCl_3_) δ (ppm) 7.384–7.309 (m, 4H), 7.309–7.237 (m, 1H), 4.521 (s, 2H), 3.786 (t, *J* = 5.0 Hz, 2H), 3.662 (t, *J* = 5.8 Hz, 2H), 1.867 (p, *J* = 5.8 Hz, 2H). ^13^C NMR (126 MHz, CDCl_3_) δ (ppm) 138.09(C), 128.47 (CH × 2), 127.73 (CH × 2), 127.66 (CH), 73.29 (CH_2_), 69.40 (CH_2_), 61.91(CH_2_), 32.12(CH_2_).

4-(Benzyloxy)-1-butanol (BzOC4OH, **3**)

Yield 67.2%. Purity 99.5%. ^1^H NMR (500 MHz, CDCl_3_) δ (ppm) 7.379–7.238 (m, 5H), 4.519 (s, 2H), 3.675–3.611 (m, 2H), 3.520 (t, *J* = 5.8 Hz, 2H), 1.766–1.626 (m, 4H). ^13^C NMR (126 MHz, CDCl_3_) δ (ppm) 138.16 (C), 128.43 (CH × 2), 127.73 (CH × 2), 127.68 (CH), 73.07 (CH_2_), 70.34 (CH_2_), 62.72 (CH_2_), 30.16 (CH_2_), 26.70 (CH_2_).

5-(Benzyloxy)-1-pentanol (BzOC5OH **4**)

Yield 67.7%. Purity 99.5%. ^1^H NMR (500 MHz, CDCl_3_) δ (ppm) 7.387–7.230 (m, 5H), 4.498 (s, 2H), 3.621 (t, *J* = 6.5 Hz, 2H), 3.479 (t, *J* = 6.5 Hz, 2H), 1.692–1.514 (m, 4H), 1.494–1.394 (m, 2H). ^13^C NMR (126 MHz, CDCl_3_) δ (ppm) 138.55 (C), 128.36 (CH × 2), 127.65 (CH × 2), 127.53 (CH), 72.93 (CH_2_), 70.30 (CH_2_), 62.77 (CH_2_), 32.49 (CH_2_), 29.45 (CH_2_), 22.43 (CH_2_).

6-(Benzyloxy)-1-hexanol (BzOC6OH, **5**)

Yield 64.7%. Purity 99.5%. ^1^H NMR (500 MHz, CDCl_3_) δ (ppm) 7.376–7. 314 (m, 4H), 7.316–7.244 (m, 1H), 4.499 (s, 2H), 3.623 (t, *J* = 6.6 Hz, 2H), 3.470 (t, *J* = 6.5 Hz, 2H), 1.662–1.521 (m, 4H), 1.455–1.316 (m, 4H). ^13^C NMR (126 MHz, CDCl_3_) δ (ppm) 138.64 (C), 128.35 (CH × 2), 127.64 (CH × 2), 127.50 (CH), 72.89 (CH_2_), 70.34 (CH_2_), 62.92 (CH_2_), 32.71 (CH_2_), 29.71 (CH_2_), 26.01 (CH_2_), 25.58 (CH_2_).

7-(Benzyloxy)-1-heptanol (BzOC7OH, **6**)

Yield 69.0%. Purity 99.5%. ^1^H NMR (500 MHz, CDCl_3_) δ (ppm) 7.399–7.245 (m, 5H), 4.500 (s, 2H), 3.628 (t, *J* = 6.7 Hz, 2H), 3.465 (t, *J* = 6.6 Hz, 2H), 1.645–1.587 (m, 2H), 1.586–1.525 (m, 2H), 1.439–1.286 (m, 6H). ^13^C NMR (126 MHz, cdcl_3_) δ (ppm) 138.68 (C), 128.34 (CH × 2), 127.62 (CH × 2), 127.48 (CH), 72.87 (CH_2_), 70.43 (CH_2_), 63.02 (CH_2_), 32.72 (CH_2_), 29.69 (CH_2_), 29.24 (CH_2_), 26.17 (CH_2_), 25.68 (CH_2_).

8-(Benzyloxy)-1-octanol (BzOC8OH, **7**)

Yield 61.0%. Purity 99.5%. ^1^H NMR (500 MHz, CDCl_3_) δ 7.396–7.230 (m, 5H), 4.499 (s, 2H), 3.623 (t, *J* = 6.7 Hz, 2H), 3.461 (t, *J* = 6.6 Hz, 2H), 1.673–1.507 (m, 4H), 1.412–1.276 (m, 8H). ^13^C NMR (126 MHz, CDCl_3_) δ (ppm) 138.70 (C), 128.34 (CH × 2), 127.63 (CH × 2), 127.47 (CH), 72.87 (CH_2_), 70.48 (CH_2_), 63.04 (CH_2_), 32.74 (CH_2_), 29.74 (CH_2_), 29.41 (CH_2_), 29.35 (CH_2_), 26.12 (CH_2_), 25.67 (CH_2_).

9-(Benzyloxy)-1-nonanol (BzOC9OH, **8**)

Yield 25.4%. Purity 99.5%. ^1^H NMR (500 MHz, CDCl_3_) δ (ppm) 7.375–7.225 (m, 5H), 4.500 (s, 2H), 3.626 (t, *J* = 6.7 Hz, 2H), 3.461 (t, *J* = 6.7 Hz, 2H), 1.666–1.564 (m, 2H), 1.564–1.509 (m, 2H), 1.404–1.203 (m, 10H).^13^C NMR (126 MHz, CDCl_3_) δ (ppm) 138.71 (C), 128.34 (CH × 2), 127.62 (CH × 2), 127.47 (CH), 72.86, (CH_2_) 70.50 (CH_2_), 63.06 (CH_2_), 32.79 (CH_2_), 29.76 (CH_2_), 29.53 (CH_2_), 29.39 (CH_2_), 29.35 (CH_2_), 26.17 (CH_2_), 25.71 (CH_2_).

### 2.3. Instrumental Analysis

GC-MS analysis was performed on an Agilent 5975C mass selective detector coupled with an Agilent 7890A (Agilent Technologies, Santa Clara, CA, USA) equipped with an HP-Innowax (30 m × 0.25 mm i.d., 0.25 μm film thickness; Agilent Technologies, Santa Clara, CA, USA). The oven temperature was programmed as 40 °C for 1 min and then raised to 250 °C at 6 °C/min, and the temperature was held for 4 min. ^1^H and ^13^C NMR (500 and 125 MHz, respectively) analysis was performed with a Varian UI500 (Agilent Technologies, Santa Clara, CA, USA) spectrometer using TMS in CDCl_3_ as an internal standard at the Korea Basic Science Institute, Seoul, Korea.

### 2.4. Nematicidal Activities of Benzyloxyalkanols against B. xylophilus In Vitro

Benzyloxyalkanols (**1**–**8**) were solubilized in 1% DMSO solution (laboratory grade, Daejung, Hwaseong, Korea) to obtain final concentrations of 1000, 500, 100, and 50 ppm using serial dilutions, and 1% DMSO solution was used as the control (mortality with the 1% DMSO solution was not different from that in the untreated groups using water). As a negative control, abamectin (Sigma–Aldrich, St. Louis, MO, USA) solution was prepared as above and final concentrations of 100, 50, 10, 5, and 1 ppm solution were tested. Benzyl alcohol in 1% DMSO solution was prepared at a final concentration of 2000 ppm, as above. The working solutions were prepared and used on the day of the experiment. Each treatment comprised three replicates, and each mortality experiment was repeated five times. Approximately 1000 nematodes were placed on each well of a 96-well cell culture plate containing 100 μL of each prepared solution and 1% DMSO solution (control). Then, the 96-well cell culture plates were maintained at 25 ± 1 °C and 40% humidity in the dark, and nematode mortality was observed at 24 h after treatment. Nematodes were considered dead if their bodies were straight and they did not move, even after transfer to clean water.

### 2.5. Extraction of Crude Protein

Crude protein of pine wood nematodes was extracted by following the method of Kang et al. [32]. Briefly, *B. xylophilus* (ca. 300 μL) specimens were transferred to a 1.5-mL tube containing 500 μL of protein extraction buffer (0.1 M Tris–HCl buffer containing 20 mM NaCl and 0.5% Triton X-100; pH 7.8) with metal beads (half of the total volume) and vigorously shaken for 3 min. To avoid protein degradation by protease, a protease inhibitor cocktail (Sigma–Aldrich) was added to the extract. The extract was centrifuged at 17,000× *g* for 15 min at 4 °C, and crude protein was separated from the cell debris and filtered through glass wool to remove the excess lipids. The concentration of crude protein isolated from the PWN was estimated with the Bradford reagent method by using a VersaMax microplate reader (Molecular Devices, Sunnyvale, CA, USA). Bovine serum albumin (BSA) was serially diluted in 0.1 M Tris–HCl buffer (pH 7.8) containing 20 mM NaCl and 0.5% Triton X-100 and was used as the standard protein for quantification.

### 2.6. Inhibitory Activity of Benzyloxyalkanols against Acetylcholinesterase (AChE) and Glutathione S-Transferase (GST)

The AChE and GST inhibitory activities of the compounds were analyzed by the methods of Ellman et al. [33] and Kang et al. [32], respectively, with modifications. Chemicals (BzOC8OH and BzOC9OH) were prepared by dilution in DMSO. One microliter of chemical and 79 μL of crude protein were mixed in a 96-well microplate. DMSO without any chemical was treated as a positive control. The concentrations of the test chemicals were 1, 0.5, 0.1 and 0.05 mg/mL. For AChE inhibition, after 10 min of preincubation, 10 μL of 10 mM acetylthiocholine iodide (ASChI) and 10 μL of 4 mM 5,5′-dithiobis (2-nitrobenzoic acid) (DTNB) were added. For GST inhibition, the substrate solution, which included 10 μL of 20 mM reduced glutathione (Sigma−Aldrich) and 10 μL of 10 mM 1-chloro-2,4-dinitrobenzene (CDNB, Sigma−Aldrich) diluted in 0.1 M Tris−HCl (pH 7.8), was added to the preincubated mixtures of proteins and the synthetic compounds. The AChE and GST inhibitory activity was estimated by measuring the *V*max for 30 min at 30 s intervals at 412 nm and 340 nm, respectively, at room temperature by using the VersaMax ELISA microplate reader. The inhibitory activity (%) was estimated as 100 ((*V*max of treatment/*V*max of control) × 100). These assays were performed in triplicate.

### 2.7. Statistics

The mortality was corrected using Abbott’s formula [34], and the corrected mortality was arcsine square-root transformed for one-way ANOVA. The means were compared and separated by the Tukey–Kramer HSD test. The LD (lethal dose) values were estimated by probit analysis with dose-response data. Statistical analysis was performed using JMP ver. 9.0.2 (SAS Institute Inc., Cary, NC, USA). Mean (± SE) values of untransformed data are reported. Untransformed data are shown.

## 3. Results and Discussion

### 3.1. Nematicidal Activities of Benzyloxyalkanols against B. xylophilus In Vitro

The nematicidal activities of benzyloxyalkanols (BzOROH) and monochamol are shown in Table 1. The mortality results of benzyloxyalkanols showed dose-dependent responses and was significantly different according to chain length. The mortality of all tested benzyloxyalkanols was higher than that of benzyl alcohol even at half concentration. BzOC8OH (**7**) and BzOC9OH (**8**) showed 100% mortality at a concentration of 1000 ppm. The mortality of benzyl alcohol was 31.0% at a concentration of 2000 ppm. As the chain length of the aliphatic portion was shortened, the mortality was weakened at all tested concentrations. At a concentration of 500 ppm, this trend was clearly shown. BzOC9OH (**9**) showed 85.1% mortality. BzOROH showed a C8–C6 carbon length in the alkyl group (**6**–**8**) and resulted in relatively mild mortality (45.4–56.0% mortality), and BzOROH showed a shorter than C6 carbon length (**1**–**4**) and exhibited weaker mortality (37.7–13.9% mortality).

Based on the dose-response data, the LC_50_ and LC_90_ values were calculated to compare toxicity (Table 2). The LD_50_ and LD_90_ of BzO8OH were higher than those of BzO9OH. The LD_50_ value of BzOC8OH was 1.6 times higher than that of BzOC9OH, and the LD_90_ value of BzOC8OH was approximately twice that of BzOC9OH. However, the toxicity of BzO8OH and BzO9OH was much weaker than that of abamectin in the terms of LD values.

Aromatic compounds in natural products are known to possess nematicidal activity. Benzylic compounds, phenylpropanoids, such as cinnamyl alcohol and cinnamic acid ester, eugenol and its analogs, and terpenoids, such as thymol and carvacrol, from essential oils (EOs) have been reported to have nematicidal activity against PWNs [18,23,35,36]. Suga et al. [37] reported that pinosylvin monomethyl ether ((*E*)-3-hydroxy-5-methoxystilbene) from pine wood extracts has nematicidal activity. This compound possesses the partial structure of phenylpropanoid and eugenol. Additionally, aromatic compounds from fungi, such as 4-hydroxyphenylacetic acid and sparassol, showed nematicidal activity against PWN [38,39]. Benzyl alcohol is also reported from plant EOs [35,40]. The mortality resulting from the benzyloxyalkanols ranged from 42.3% to 100% at a concentration of 1000 ppm, and they showed nematicidal activity.

Recently, Kim et al. [29] reported the nematicidal activity of the homologues of 2-(1-undecyloxy)-1-ethanol against PWN, and modification of the hydroxy group of alkanols (-OH) to the ɷ-hydroxyalkyloxy group (-OROH) increased their nematicidal activity against PWN [29,41]. In this study, the same modification was performed with benzyl alcohol, and their nematicidal activity against PWN was tested. Benzyloxyalkanols showed higher nematicidal activity than benzyl alcohol even at a half concentration of benzyl alcohol. From these results, it can be concluded that the modification of the hydroxy group to the ɷ-hydroxyalkyloxy group could increase nematicidal activity. There are many reports about aromatic compounds showing higher nematicidal activity than benzyl alcohol. These aromatic compounds possess phenolic hydroxy groups and phenylallyl groups [18,35,42]. The increase in nematicidal activity of such compounds could be expected by modification of the hydroxy group to the ɷ-hydroxyalkyloxy group.

As the carbon chain length of the alkyl group in benzyloxyalkanols increased, the nematicidal activity increased. Similar trends were also reported for alkyloxyethanols and aliphatic alcohols [29,41]. Not only the chain length of alkyl groups in alkyloxyethanols but also the chain length of ɷ-hydroxyalkyloxy groups in benzyloxyalkanols seemed to be responsible for the nematicidal activity. The relationship between the structure and activity of pine wood nematodes has been scarcely reported. The relationship of the toxicity and linear carboxylic acids with C_4_-C_10_ carbon atom chains suggested that structural characteristics would allow for easy transfer of the compound through the insect cuticle [43]. Li et al. [44] suggested that the steric hindrance of chain analogs affects the toxicity of aliphatic isothiocyanate. However, the relationship between the nematicidal activity and the structure of benzyloxyalkanols remains to be resolved.

### 3.2. Inhibitory Activity of Benzyloxyalkanols against Acetylcholinesterase (AChE) and Glutathione S-Transferase (GST)

The PWN AChE and GST inhibition rates were determined with the two compounds, BzOC8OH (**7**) and BzOC9OH (**8**), which showed the highest mortality among the tested compounds. In the AChE and GST inhibitory assays, two compounds did not show any inhibitory activities at any tested concentration (Table 3).

The management of pests relies mainly on the use of insecticides, and the continuous use of insecticides may result in the development of resistance in pests. To overcome or suppress the resistance of a pest against pesticides, it is recommended that active compounds with different modes of action be used [45]. There are few reports on the modes of action of aromatic compounds against PWN. Cha et al. [19] reported that naphthoquinones generate reactive oxygen species that cause oxidative stress in *B. xylophilus*. Rajasekharan et al. [46] reported that 5-iodoindole, like abamectin, rigidly binds to the active sites of the GluCL receptor by molecular docking assay and suggested that this binding is crucial for maintaining the open pore structure of the GluCL complex. 5-Iodoindole also induced diverse phenotypic deformities in nematodes, including abnormal organ disruption/shrinkage and increased vacuolization. The AChE inhibitory activity of phenylpropanoid and eugenol analogs was tested by Kang et al. [47]. Isoeugenol and *o*-anisaldehyde showed approximately 50% inhibitory activity against AChE at a concentration of 1000 ppm, while other eugenol analogs showed less than 10% inhibitory activity. Although not aromatic, the inhibitory activity of aliphatic compounds against PWN, AChE and GST were estimated to correspond to the mode of action [32]; some aldehyde compounds showed >70% AChE inhibition and alkanoic acid showed approximately 40% GST inhibition. However, the compounds that showed enzyme inhibitory activity did not coincide with those with the best nematicidal activity. In this study, BzOC8OH and BzOC9OH showed relatively higher nematicidal activity than the other tested compounds; however, they showed no or little inhibition of the AChE and GST of PWN. This result suggests that AChE and GST may not be the targets of benzyloxyalkanols. For the safe practical use of benzyloxyalkanols as nematicides, the other mode of action of benzyloxyalkanols should be investigated.

## 4. Conclusions

In this study, benzyloxyalcohols were investigated for their nematicidal activities against *B. xylophilus* for the first time. Our results indicated that benzyloxyalcohols could be alternative nematicidal agents and that modification of the hydroxy group to the ɷ-hydroxyalkyloxy group could increase their nematicidal activity. However, further studies, including elucidation of the mode of action, are necessary for developing nematicidal agents.

## 5. Patents

Kim J., Lee S., 2019. Composition for controlling pine wood nematodes containing benzyloxyalcohol. Korea Patent Application Publication, Application No. 10-2019-0156768.

## Figures and Tables

**Table 1:** Nematicidal activity of benzyloxyalkanols.

Treatment	Mortality at Each Concentration (%, Mean ± SE)				
	2000 ^1^	1000	500	100	50
Benzyl alcohol	31.0 ± 3.9	-	-	-	-
BzOC2OH (1)	-	30.6 ± 4.2 ^d^	13.9 ± 6.3 ^e,f^	2.5 ± 0.6 ^e^	-
BzOC3OH (2)	-	43.3 ± 4.6 ^c,d^	25.9 ± 2.5 ^d,e^	4.9 ± 0.7 ^e^	-
BzOC4OH (3)	-	53.2 ± 4.2 ^c^	33.0 ± 4.0 ^c,d^	5.3 ± 0.4 ^d,e^	-
BzOC5OH (4)	-	57.4 ± 4.7 ^b,c^	37.7 ± 3.0 ^c,d^	8.8 ± 1.1 ^c,d,e^	-
BzOC6OH (5)	-	71.5 ± 7.5 ^b^	45.4 ± 3.0 ^b,c^	12.8 ± 1.2 ^c,d^	-
BzOC7OH (6)	-	60.2 ± 5.3 ^b,c^	47.6 ± 3.2 ^b,c^	16.2 ± 4.3 ^c^	-
BzOC8OH (7)	-	100 ^a^	56.0 ± 4.3 ^b^	28.9 ± 2.9 ^b^	5.2 ± 0.5 ^a^
BzOC9OH (8)	-	100 ^a^	85.1 ± 1.9 ^a^	39.2 ± 2.4 ^a^	5.0 ± 0.5 ^a^
Control	-	0 ^e^	0 ^f^	0 ^g^	0 ^b^
Statistical value	*-*	*F*_8,36_ = 159.1 *p* < 0.001	*F*_8,36_ = 67.0 *p* < 0.001	*F*_8,36_ = 42.43 *p* < 0.001	*F*_2,12_ = 48.83 *p* < 0.001

^1^ ppm. Within the same column, different uppercase letters indicate statistical differences among the treatment, as measured by one-way ANOVA analysis followed by Tukey’s test (*p* < 0.05).

**Table 2:** LD_50_ and LD_90_ values of benzyloxyheptanol (**7**), bezyloxyoctanol (**8**), and abamectin.

Treatment	LD_50_ (95% CL) ^1^	LD_90_ (95% CL)	χ^2^ (df)
BzOC8OH (**7**)	246.1 (228.5–265.2)	1004.6 (893.1–1145.6)	193.1 (18)
BzOC9OH (**8**)	158.0 (81.4–168.9)	490.0 (444.9–545.3)	71.5 (18)
Abamectin	1.7 (1.51–1.94)	11.8 (10.2–13.9)	147.1 (18)

^1^ LC_50_ and LC_90_ values are in ppm and 95% CL are confidence limits.

**Table 3:** Acetylcholinesterase (AChE) and glutathione *S*-transferase (GST) inhibitory activity of benzyloxyheptanol (**7**) and benzyloxyoctanol (**8**).

Treatment	Concentration (ppm)	Inhibitory Activity (%)	
		AChE	GST
BzOC8OH (**7**)	50	−0.22	−3.45
	100	1.37	−2.86
	500	2.57	−3.64
	1000	0.19	−5.24
BzOC9OH (**8**)	50	0.17	0.01
	100	−0.24	0.45
	500	−0.01	−2.60
	1000	0.60	−6.74
